# Metabolic Plasiticy in Cancers—Distinct Role of Glycolytic Enzymes GPI, LDHs or Membrane Transporters MCTs

**DOI:** 10.3389/fonc.2017.00313

**Published:** 2017-12-20

**Authors:** Maša Ždralević, Ibtissam Marchiq, Monique M. Cunha de Padua, Scott K. Parks, Jacques Pouysségur

**Affiliations:** ^1^Institute for Research on Cancer and Aging (IRCAN), CNRS, INSERM, Centre A. Lacassagne, University Côte d’Azur, Nice, France; ^2^Medical Biology Department, Centre Scientifique de Monaco (CSM), Monaco, Monaco

**Keywords:** cancer, CRISPR-Cas9, glycolysis, immune response, lactic acid, metabolism, oxidative phosphorylation, pentose phosphate pathway

## Abstract

Research on cancer metabolism has recently re-surfaced as a major focal point in cancer field with a reprogrammed metabolism no longer being considered as a mere consequence of oncogenic transformation, but as a hallmark of cancer. Reprogramming metabolic pathways and nutrient sensing is an elaborate way by which cancer cells respond to high bioenergetic and anabolic demands during tumorigenesis. Thus, inhibiting specific metabolic pathways at defined steps should provide potent ways of arresting tumor growth. However, both animal models and clinical observations have revealed that this approach is seriously limited by an extraordinary cellular metabolic plasticity. The classical example of cancer metabolic reprogramming is the preference for aerobic glycolysis, or Warburg effect, where cancers increase their glycolytic flux and produce lactate regardless of the presence of the oxygen. This allows cancer cells to meet the metabolic requirements for high rates of proliferation. Here, we discuss the benefits and limitations of disrupting fermentative glycolysis for impeding tumor growth at three levels of the pathway: (i) an upstream block at the level of the glucose-6-phosphate isomerase (GPI), (ii) a downstream block at the level of lactate dehydrogenases (LDH, isoforms A and B), and (iii) the endpoint block preventing lactic acid export (MCT1/4). Using these examples of genetic disruption targeting glycolysis studied in our lab, we will discuss the responses of different cancer cell lines in terms of metabolic rewiring, growth arrest, and tumor escape and compare it with the broader literature.

## Introduction

As opposed to normal, differentiated cells, which under aerobic conditions metabolize glucose mainly *via* oxidative phosphorylation (OXPHOS), cancer cells largely favor glycolytic pathway and subsequent lactate^1^ formation for their energy production, regardless of oxygen availability. Warburg first observed this metabolic peculiarity of cancer cells (1) and postulated not only that cancer cells have damaged respiration and excessive glycolysis but also that the shift of energy metabolism from aerobic to anaerobic is actually the cause of cancer (1). According to Warburg, the tumor is initiated by irreversible damage to respiration and persists because of increased anaerobic metabolism, which compensates energetically for the failure of respiration (1). However, today we know that many cancer cells have healthy mitochondria (2) and rely partly on oxidative metabolism (3), whereas fermentative glycolysis remains the “preferred” pathway by most hypoxic and rapidly growing tumors (4–6).

Following these pioneering studies, the field of cancer metabolism has been in a shadow of cancer genetics, which prevailed for decades, after the discovery of the role of oncogenes and tumor-suppressor genes in cancer. However, in the late 1990s, it was shown that lactate dehydrogenase A (*LDHA*) is a direct c-Myc-responsive gene (7), followed later on by the discovery that c-Myc and HIF-1 complementary induce all glycolytic enzymes with a concomitant inhibition of the pyruvate oxidation (8), reviving interest in connecting oncogenes and altered metabolism (4). At this time, altered metabolism was seen only as a consequence of oncogenic activation, since serum growth factors known to rapidly activate metabolism in the early 1970s (9) were shown to induce c-Myc. Interestingly, it was shown only later that loss-of-function mutations of the TCA cycle enzymes succinate dehydrogenase (10) and fumarate hydratase (11) were implicated in pathogenesis of several hereditary forms of cancer. These mutations in tumor-suppressor genes encoding for important metabolic enzymes raised the possibility that under certain conditions, altered metabolism could be the cause, not the effect, of cancer transformation (12).

**Figure 1:**
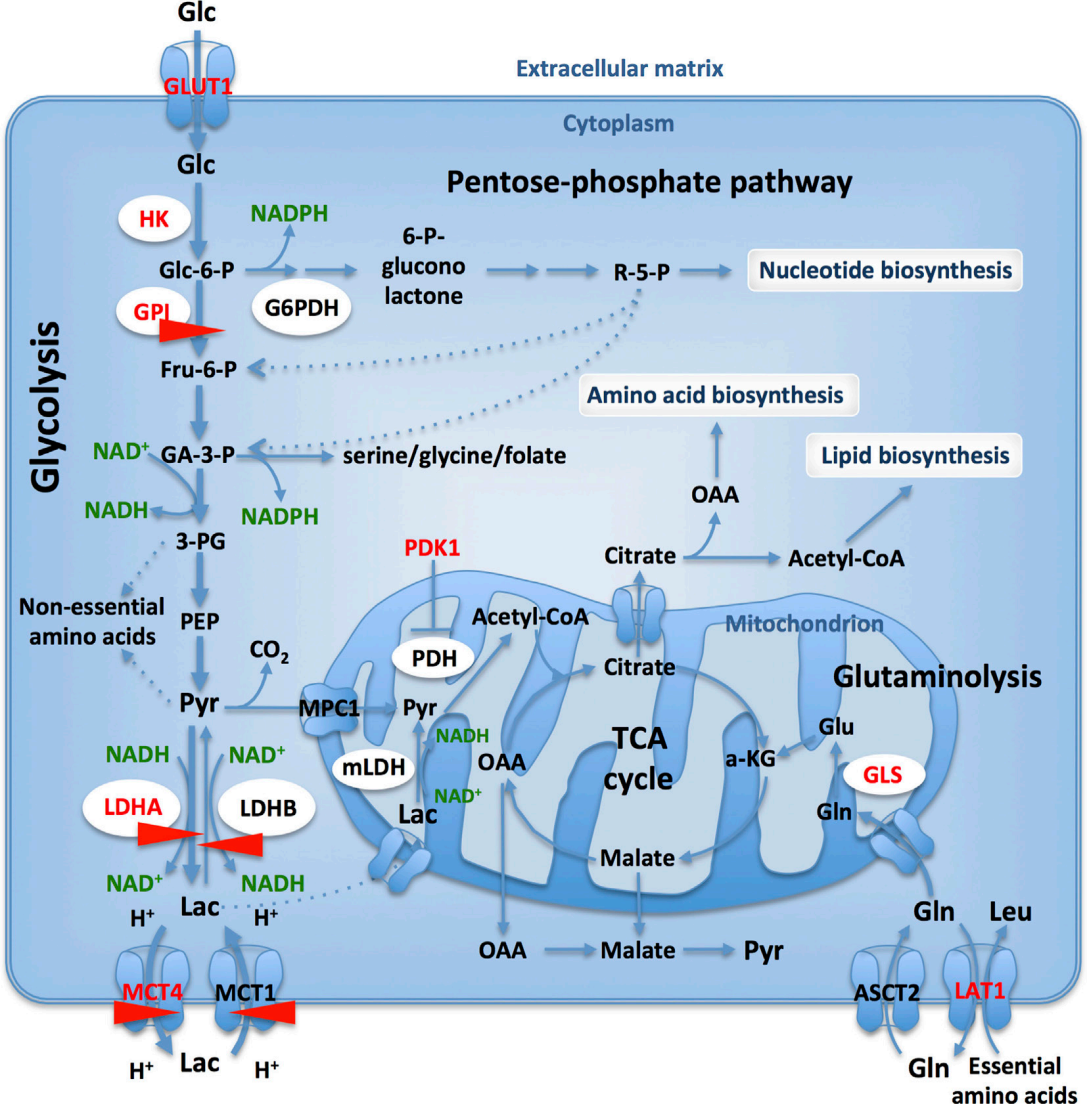
Glucose and glutamine catabolism provide tumor cells with biosynthetic precursors. Glucose transport and glycolytic flux are accelerated in cancer cells, when compared to normal cells, due to increased expression of appropriate transporters and enzyme isoforms. Glucose-6-phosphate dehydrogenase (G6PDH) shunts G6P from the glycolysis into the oxidative branch of pentose phosphate pathway (PPP). Intermediates from glycolysis and TCA cycle replenish biosynthetic pathways to produce macromolecules (nucleic acids, lipids, and proteins) necessary for cell proliferation. Only those transporters and enzymes relevant to the text are shown: GLUT1, glucose-6-phosphate isomerase, lactate dehydrogenase A (LDHA)/-B, MCT1/4. HIF- targets are in red and CRISPR-Cas9 targets studied in our lab are identified with red arrows.

Even if seemingly counterintuitive, given the much lower ATP yield from glycolysis with respect to the OXPHOS, this reprogramming of energy metabolism is thought to support large-scale macromolecule biosynthesis, necessary for rapid proliferation and growth (5, 6, 13) (Figure 1). Metabolic rearrangements are a feature of almost all cancer cells, which enables them to adapt to constantly changing conditions in nutrient microenvironment thereby promoting their aberrant proliferation. Aerobic glycolysis (Warburg effect) is just one component of the metabolic transformation, together with the reverse Warburg effect (14), metabolic symbiosis (15) and addiction to glutamine metabolism (16).

In this mini-review, we report the tumor growth consequences of re-routing fermentative glycolysis by genetic disruption at three key levels studied in our lab: glucose-6-phosphate isomerase (GPI), lactate dehydrogenase (LDHA and B isoforms), and at the level of export of lactic acid [monocarboxylate transporter (MCT) isoforms]. We discuss their responses in terms of metabolic rewiring, growth arrest, or tumor escape and compare it with a broader literature.

## Aerobic Glycolysis and the Control of the Metabolic Switch

Despite the remarkable genetic and phenotypic tumor heterogeneity, a specific set of signaling pathways appear to support the altered metabolic processing of glucose. Indeed, there is a dual set of universal mitogenic pathways: Ras-Raf-ERK and PI3K-AKT activated by growth factors/hormone receptor tyrosine kinases and G protein-coupled receptors. ERKs and AKTs protein kinases synergize in controlling growth and metabolism through activation of the master protein kinase (mTORC1). In cancer, oncogenes and tumors suppressors constitutively activate these mitogenic pathways to modify metabolism, nutrient, and oxygen sensing through c-Myc and HIF-1 (17–19). Regulation of cancer cells’ metabolic rearrangements by oncogenes and tumor suppressors is complex and beyond the scope of this short review, but the fact that numerous pathways converge on glucose and glutamine reflects their central importance for energy metabolism.

The avidity of cancer cells for glucose is reflected by the upregulation of glucose transporters and clinical exploitation of the accumulation of radioactive ^18^F-deoxyglucose is identified by positron emission tomography. Once inside the cell, glucose is metabolized by glycolysis, a pathway embedded in a complex metabolic network, directly providing precursors for nonessential amino acids (20) and through branching to the oxidative arm of pentose phosphate pathway (PPP), nucleotides (20) (Figure 1). Furthermore, NADPH is regenerated in the PPP and by the serine, glycine/C1-carbon synthesis glycolytic bypass thus contributing to reductive biosynthesis and redox homeostasis (21). As such, branching of the glycolytic pathway is strictly regulated at several different steps (22).

Recognition that the oncogenic activation leads to increased glycolysis (23), together with clinical evidence that correlated cell metabolism with cancer outcome, prompted many studies toward strategies to inhibit glucose metabolism in cancer (24, 25). In fact, some of the first metabolic anticancer therapies developed remain effective agents in clinic today, such as antifolate drugs and l-asparaginase (25). 2-deoxy-glucose (2-DG) has been recognized as a glycolysis inhibitor since the 1950s (26, 27), primarily by competitively inhibiting GPI (26, 28). However, 2-DG also inhibits glucose transport (29), hexokinase (HK) activity (30, 31) and the multiple points of action and its high toxicity have prevented its use in the clinic (32, 33).

## Glucose-6-Phosphate Isomerase (GPI)

Glucose-6-phosphate isomerase (d-glucose-6-phosphate aldose-ketose-isomerase; EC 5.3.1.9) is a housekeeping cytosolic enzyme that plays a key role in glycolytic and gluconeogenic pathways, catalyzing the interconversion between G6P and fructose-6-phosphate (Figure 1). Its expression is induced by c-Myc (34) and HIF-1 (35, 36) and is increased in many cancers (37). GPI has also been described as a secreted multifunctional complex protein that could act as a cytokine under the name autocrine motility factor (38). However, this notion requires further confirmation.

In our lab a complete genetic ablation of *GPI* expression was accomplished by using CRISPR/Cas9 in two aggressive cancer cell lines, human colon adenocarcinoma (LS174T) and mouse melanoma (B16-F10) (39). Both *GPI*-mutant cell lines had no detectable GPI enzymatic activity, suppressed completely lactic acid secretion and grew by reprogramming their bioenergetic metabolism to OXPHOS (39). Surprisingly, in contrast to previous pharmacological inhibition studies (29, 37), *GPI*-KO cells growth was only reduced by twofold in normoxia with ATP produced by OXPHOS being sufficient to maintain their growth and viability. However, the growth rate of *GPI*-KO cells was severely reduced in hypoxia (1% O_2_) while cells remained viable. Interruption of the glycolytic flow by *GPI*-KO increases the intracellular G6P pool, which in turn was proposed to elicit a short-term inhibition of HK and a long-term inhibition of glucose transport (40, 41). Indeed, we found that both *GPI*-KO cell lines had decreased GLUT1 expression, as well as induction of thioredoxin-interacting protein expression, a strong negative regulator of glucose uptake (42). We showed that increased OXPHOS dependence of *GPI*-KO cells made them extremely sensitive to inhibitors of the respiratory chain complexes, such as phenformin and oligomycin (39), in line with the findings of Pusapati et al. (37). Therefore, we speculate that pharmacological inhibition of tumor growth at the level of GPI was effective mainly because of the multiple targets of 2-DG.

**Figure 2:**
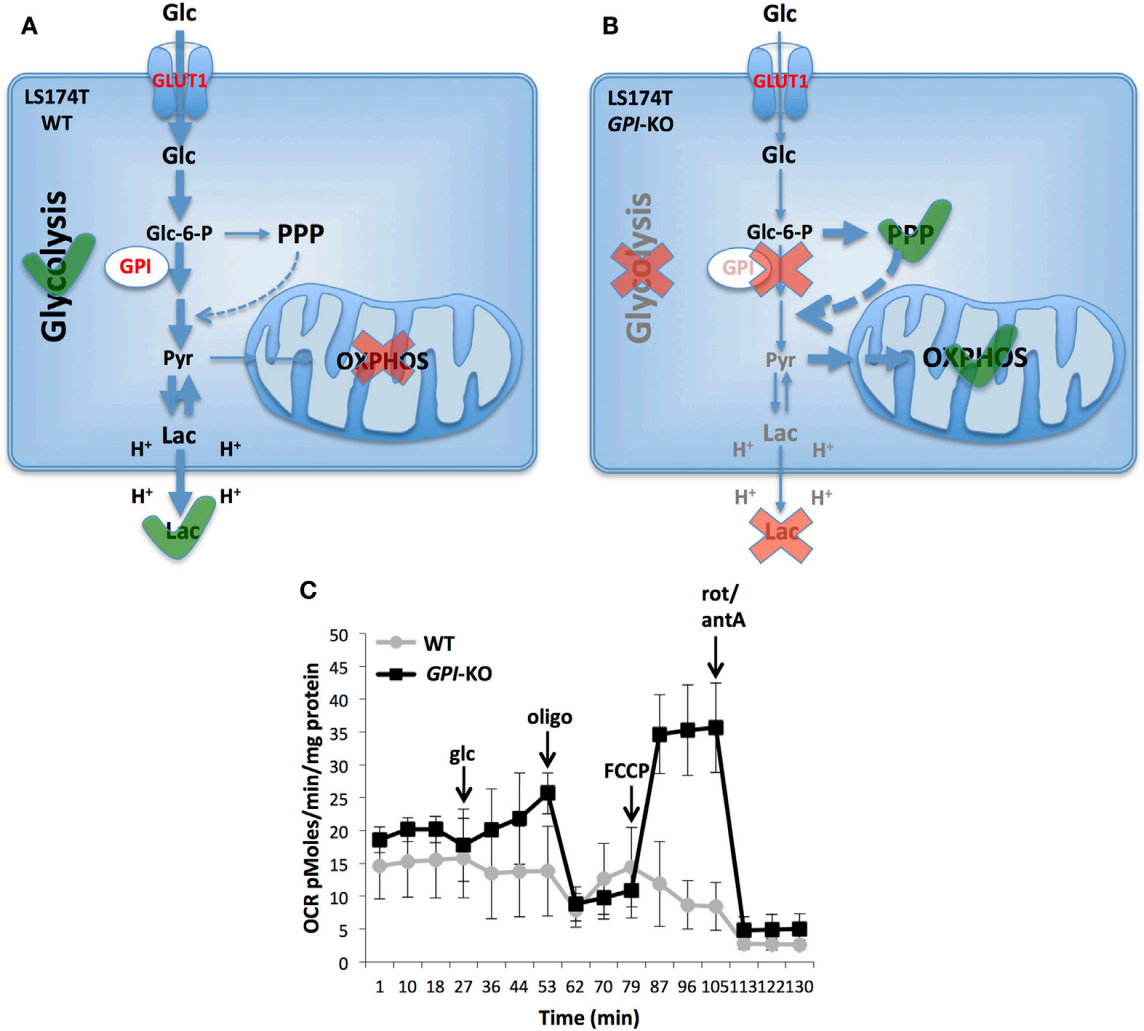
Metabolic reprogramming in glucose-6-phosphate isomerase (*GPI*)-KO cells. A switch from glycolytic metabolism to oxidative phosphorylation (OXPHOS) caused by the complete *GPI* disruption is shown. LS174T WT cells are highly glycolytic and do not use mitochondria for ATP production **(A)**. Contrarily, cells survive *GPI* disruption by re-activating pentose phosphate pathway (PPP) and OXPHOS **(B)**. Oxygen consumption rate (OCR) of LS174T WT and *GPI*-KO cells was evaluated with Seahorse XF24 bioanalyzer **(C)**. The mean ± SEM is representative of four independent experiments performed in quadruplicate. The figure is adapted from Ref. (39). Glc, glucose, oligo, oligomycin, FCCP, carbonyl cyanide-4-(trifluoromethoxy) phenylhydrazone, rot, rotenone, antA, antimycin A.

In conclusion, we showed that complete suppression of glycolysis in two aggressive cancer cell lines slowed, but did not prevent *in vivo* tumor growth, in line with the findings of Pouysségur et al. (40) and Pusapati et al. (37). Particularly striking is the LS174T cell line that is highly glycolytic and almost does not respire under normal conditions and is capable to achieve strong re-activation of OXPHOS when challenged by *GPI* ablation (Figure 2). Consequently, as shown with inducible shRNAs against *GPI*, the growth was significantly reduced only in combination with mTORC1 or OXPHOS inhibition (37). This remarkable metabolic plasticity of cancer cells revealed as well on several other cell lines (37) poses a big challenge for anticancer therapies targeting metabolism.

## Lactate Dehydrogenase (LDH) Isoforms

Lactate dehydrogenase [(*S*)-lactate:NAD^+^ oxidoreductase; EC 1.1.1.27] is a family of NAD^+^-dependent enzymes that catalyze the interconversion between pyruvate and lactate, with concomitant oxidation/reduction of the cofactor (NADH/NAD^+^). LDH is a homo- or hetero-tetramer assembled from two different subunits: M and H, encoded by two separate genes, *LDHA* (M) and *LDHB* (H), respectively. A third subunit, LDHC, encoded by a separate *LDHC* gene, is expressed only in testes and sperm and is probably a duplication of the *LDHA* gene (43). LDH tetramers form at least six isoenzymes that differ in electrophoretic mobility, *K*m for pyruvate and lactate, immunological characteristics, thermal stability and inhibition by coenzyme analogs or excess pyruvate (44). The existence of mitochondrial LDH was shown in prostate cancer cells (45), and human hepatocellular carcinoma cells (46). Mitochondrial metabolism of lactate results in export of oxaloacetate, malate, and citrate outside mitochondria, therefore having an anaplerotic role (Figure 1) (46). In this mini-review, we will focus on the cytosolic LDH and refer readers to excellent reviews on this topic (47, 48).

### LDHA

Lactate dehydrogenase A (LDH-5, or LDHA4) is composed of four LDHA subunits and has the lowest *K*m for pyruvate of the LDH isoforms and catalyzes pyruvate reduction to lactate, the final step of the glycolysis, with concomitant regeneration of NAD^+^ molecules, required for glycolysis to proceed. LDHA is located mainly in the cytoplasm, but it has also been found to bind single-stranded DNA in the nucleus (49). LDHA has been recognized as a valuable predictive/prognostic marker; its overexpression is associated with cancer invasiveness, and elevated serum lactate levels correlate with poor prognosis and resistance to chemo- and radiotherapy (50). LDHA expression is regulated by c-Myc (7), HIF-1 (51, 52), and micro-RNA miR-34a (53). The key role of LDHA in maintaining the Warburg phenotype in cancer cells was confirmed by several reports of LDHA inhibition or knockdown severely diminishing tumorigenicity in breast, lung, liver, lymphoma, and pancreas cancers (54–58). Decreased LDHA activity resulted in stimulation of OXPHOS and mitochondrial oxygen consumption and decrease of mitochondrial membrane potential (54) and increased apoptosis *via* ROS production (56–58). These data, together with the fact that LDHA deficiency has no serious consequences under normal conditions made LDHA a very attractive target for the anticancer therapy. Many LDHA inhibitors shown to suppress tumor growth *in vitro* and *in vivo* were developed by major pharmaceutical groups, but with moderate selectivity, particularly of those targeting the dinucleotide binding site common to many enzymes (50). These inhibitors were more powerful in combination with other therapies, but none have reached the stage of clinical trials (50). Recently, Genentech group described a novel LDHA inhibitor, GNE-140, capable of inhibiting both isoforms with nanomolar potency (59). Their work showed that predominantly glycolytic cell lines were more sensitive to LDHA inhibition, while cell lines relying more on OXPHOS were inherently resistant (59), and in these cells the combination of LDHA inhibition with OXPHOS inhibitors was synthetically lethal (59). However, GNE-140 was unable to inhibit tumor growth *in vivo*, alone or in combination with phenformin, due to its rapid clearance.

Conversely, our work with *LDHA*-KO cells in LS174T and B16 cell lines shows that LDHA is dispensable for *in vitro* tumor growth, both in normoxia and in hypoxia. These cells were still able to catalyze pyruvate conversion to lactate. Although reduced, this activity was sufficient to drive glycolysis and lactate production, which was only moderately decreased with respect to WT cells (60, 61). *LDHA*-KO cells moderately stimulated OXPHOS and, therefore, were more sensitive to respiratory chain inhibitors. However, residual LDH activity present in these cells, which we argue is due to the activity of the LDHB isoform, was sufficient to sustain cell growth and viability. Thus, we argue that most of the alterations due to LDHA inhibitors shown so far were due to off-target effects and not a specific decrease in LDHA activity. Similar results were observed in a study of *LDHA* silencing in breast cancer cell line, where stable *LDHA* knock down did not affect cell viability, lactic acid production, glucose consumption, or ATP (62). These cells contained twice as much LDHB isoform, again supporting the possibility of the LDHB isoform catalyzing the reverse reaction.

### LDHB

LDHB is composed of four B subunits and catalyzes lactate oxidation to pyruvate, coupled with NADH formation. An increasing number of studies investigated the role of LDHB in several subtypes of cancer, but its role remains elusive and poorly characterized. LDHB was found to be positively regulated by the RTK–PI3K–AKT–mTOR pathway both in immortalized mouse cell lines and human cancer cells (63). Its expression was stimulated by signal transducer and activator of transcription STAT3, a key tumorigenic driver in many cancers (63). Furthermore, LDHB was found to be upregulated in triple-negative breast cancer, KRAS-dependent lung adenocarcinoma, maxillary sinus squamous cell cancer as well as in osteosarcoma and correlated with poor patient outcome (64–67). *LDHB* knock down inhibited cell growth, proliferation, and invasion and the loss of LDHB was shown to arrest tumor growth *in vitro* an *in vivo* (64, 66, 67). This is in line with the “reverse Warburg effect,” proposing that stromal or cancer cells undergo aerobic glycolysis and produce lactate, which is then taken up by MCT1 to fuel oxidative cells *via* LDHB-catalyzed conversion to pyruvate (14, 68, 69). Indeed, MCT1 expression was found to correlate with high LDHB expression in TNBC (64).

Conversely, other studies found LDHB overexpression to be correlated with better prognosis (70), and accordingly, loss of *LDHB* expression was associated with metastatic progression (71). The underlying mechanism seems to involve *LDHB* promoter hypermethylation and consequent gene silencing at the transcriptional level (71), but exactly how loss of *LDHB* contributes to tumor progression is not clearly understood.

In our lab, *LDHB* gene knockout by CRISPR/Cas9 in LS174T and B16 cells did not significantly alter their growth and viability in normoxia or hypoxia (61). As expected, *LDHA/B*-DKO cells retained the ability to convert lactate into pyruvate by LDHA isoenzyme. Because our *LDHA*-KO cells were still capable to produce and secrete measurable levels of lactic acid we genetically disrupted the two *LDH* isoforms (*LDHA/B*-DKO) in LS174T and B16 cell lines. LDH enzymatic activity in both directions was completely abolished in these cells. As a consequence, they showed a distinctive phenotype—growth reduction, absence of glycolysis, and no lactic acid secretion, neither in normoxia nor in hypoxia (1% O_2_). Furthermore, in order to overcome the imposed glycolytic blockade, these double *LDHA/B*-DKO cells re-directed their metabolism toward OXPHOS and relied on it for viability and growth. In contrast to wild-type or single *LDH*-KO cells, the double *LDHA/B*-DKO cells died rapidly in response to mitochondrial respiratory chain inhibitors, such as phenformin and oligomycin (*in submission*).

These findings, based on a genetic approach, demonstrate that both LDHA and B contribute to fermentative glycolysis (Warburg effect) and because of the bioenergetics metabolism re-routing these two enzymes are dispensable for tumor growth. In contrast, these results point that most of the LDHA inhibitors used so far, with the exception of GNE-140 from Genentech, inhibited tumor growth due to off-target effects.

## MCT1 and MCT4

Lactic acid, the end product of fermentative glycolysis abundantly released by cancer cells, has a strong impact in tumor microenvironment (72, 73). It can function as an oxidizable fuel, gluconeogenetic precursor and a source of TCA cycle intermediates (46, 74, 75). In addition, it is an antioxidant promoting angiogenesis, migration (76), and its contribution to tumor acidosis was reported to blunt tumor-immune response by T and NK cells (60). Lactic acid is exported/imported in cells by a family of four reversible MCTs [for review, see Ref. (77)]. MCTs as H^+^/Lactate^−^ symporters facilitate net lactic acid exchange across the plasma membrane, whose direction depends on the concentration gradients of protons and monocarboxylate (77). Increasing experimental evidences support the cell–cell and intracellular lactate shuttles hypothesis proposed by Brooks (48), thus lactate is continuously formed and consumed in different cells under fully aerobic physiological conditions (48). MCT1 facilitates lactate and pyruvate transport, it is induced by c-Myc and expressed virtually in all cells. In contrast, MCT4 is an efficient lactate exporter induced by hypoxia and expressed in glycolytic tissues and cancer cells (77). Both MCT1 and 4 need assistance from the chaperone CD147 or basigine (BSG) to express active transporters at the plasma membrane.

Several reports from Baltazar’s group (78–80) have shown that increased expression of MCT1 and MCT4 are associated with a poor prognosis in several types of human cancer, such as neuroblastoma, colorectal carcinoma, gastrointestinal stromal tumors, and prostate cancer. In parallel, our group, exploring pHi-regulating systems as putative anticancer targets in hypoxic tumors (81, 82), developed an interest in blocking lactic acid export. Pharmacological blockage with the specific AstraZeneca MCT1/2 inhibitor (AZD3965) was very efficient in arresting growth of tumors expressing only MCT1, like in transformed fibroblasts (83) or neoplastic B cells (84). However, it became clear that most aggressive cancers express both isoforms, like in colon adenocarcinoma, glioblastoma or non-small cell lung cancer. In these cancer types, genetic disruption of the chaperone (BSG), with zinc finger nucleases, reduced lactic acid export by 70–80%, an action sufficient to re-activate OXPHOS and maintain tumor growth (85). These tumor cells behaved like *GPI*-KO or *LDHA/B*-DKO with growth arrest and loss of cell viability induced by inhibitors of mitochondrial respiration (85, 86). However, pharmacological inhibition of MCT1 combined with a *MCT4*-KO was able to slow considerably *in vitro* growth and *in vivo* tumor xenografts (85, 86). We also confirmed that dual pharmacological inhibition of MCT1 and MCT4 considerably reduced cell growth. Removal of the inhibitors after a week allowed cells to form colonies, indicating a cytostatic, not cytotoxic effect induced by lactic acid sequestration in response to MCTs blockade.

## Conclusion

Comparing the three independent approaches of interrupting the glycolytic flux, we reach a common consensus and a strong divergence. Genetic disruption of *GPI, LDHA/B*, or *MCT1/4* leads to re-activation of OXPHOS with tumor growth maintenance but increased sensitivity to mitochondrial inhibitors. The case of MCT1/MCT4 is interesting because the phenotype depends on the value of MCT suppression. Partial MCT suppression reached in *BSG*-KO cells, growth is maintained; total block with dual inhibition by AZD compounds, growth is compromised due to intracellular acidification.

Finally, targeting tumor metabolism *via* anti-glycolytic thera-pies remains an attractive therapeutic approach (82, 87), especially in combination with the inhibition of mitochondrial pathways, but it will have to be precisely administered in order to spare normal cells and limit toxicity (82).

## Author Contributions

MŽ isolated and characterized LDHA and B mutant cells, IM isolated and characterized MCT and BSG mutant cells and MP isolated and characterized GPI-mutant cells. SP helped with manuscript editing. MŽ and JP designed the project and wrote the manuscript.

## Conflict of Interest Statement

The authors declare that the research was conducted in the absence of any commercial or financial relationships that could be construed as a potential conflict of interest.

## Footnotes

^1^The authors refer to l-lactate metabolism in this mini-review.
